# Ultraviolet-activated clamshell hydroxyapatite-substituted palladium in the photoreduction of methyl orange water pollutant

**DOI:** 10.55730/1300-0527.3558

**Published:** 2023-03-27

**Authors:** Anis Liyana AZHAR, Azman MA’AMOR, Nurhidayatullaili MUHD JULKAPLI, Nor Asrina SAIRI, Aina Sofea NORAIZAT

**Affiliations:** 1 Department of Chemistry, Faculty of Science, University of Malaya, Kuala Lumpur, Malaysia; 2 Nanotechnology and Catalysis Research Centre (NANOCAT), Institute for Advanced Studies, University of Malaya, Kuala Lumpur, Malaysia

**Keywords:** Photocatalyst, green chemistry, biomaterials, metal-doped, synthetic dyes

## Abstract

This study focuses on the modification of natural hydroxyapatite (HAp) derived from clamshell by impregnation with palladium (Pd) at different pH in wet precipitation method to produce photoactive green materials for the degradation of synthetic dyes. It was found that, at pH 10, Pd has been successfully impregnated into HAp lattice with Ca/P ratio of 1.77 and particle distribution size range of 40–470 nm. The impregnation has resulted in the band gap of Pd/HAp at 3.19 eV, as calculated using Tauc’s plot from the UV-Vis spectroscopy data of the Pd/HAp. Next, the photocatalytic activities of Pd/HAp were carried out with methyl oranges (MO) as models of water pollutants under UV irradiation. The photodegradation efficiency of the catalyst reached the optimum value of 41.63%, 48.17%, and 43.64% after 120 min of continuous UV irradiation for pH values 8, 10, and 11.5 of Pd/HAp samples, respectively. This study opens a new paradigm in using naturally derived materials as photocatalysts in the reduction of persistent water pollutants at a low cost and green sustainable approach.

## 1. Introduction

Standard industrial wastewater treatment procedures such as flocculation, ion exchange, reverse osmosis, adsorption, and biological digestion have been developed due to the stability of advanced dyes. However, as a consequence of the generation of secondary pollutants and the partial breakdown of dye molecules, these procedures are ineffectual [1–3]. In this context, photocatalysis has garnered considerable attention in the last few years. Photocatalysis is an advanced oxidation process (AOP) in which photons of sufficient energy impact with the surface of the photocatalyst [2, 4]. With that in mind, palladium (Pd) is chosen as the photocatalyst in this work. Pd was predicted to absorb UV and visible irradiation owing to interband transition since it lacked a localized surface plasmonic resonance (LSPR) effect [5, 6].

Hydroxyapatite (HAp), a primary mineral element of mammalian hard tissues with the chemical formula Ca_10_(PO_4_)_6_(OH)_2_, has been widely employed as a covering material for metallic implants [7–9]. Several researchers, however, have lately sought to synthesize HAp from natural sources and wastes such as bovine bones, clamshells, and eggshells due to their abundant availability with low cost of production [10–12]. Indeed, because of the expensive cost of ingredients, hazardous chemicals utilized, and sophisticated procedures, traditional methods of making HAp are not practicable for large-scale production. They are also time-consuming and need precise control of the drying and sintering reactions.

In recent years, HAp has been reported as a good photocatalyst in degrading dyes (e.g., methylene blue (MB) [13–16], methyl orange (MO) [17, 18], methyl violet [19], remazol brilliant blue (RBBR) [20], and Congo red [21] ) under UV light irradiation. Begum et al. [20] reported that the synthesis of HAp through wet chemical precipitation process can be used to degrade RBBR under UV irradiation for 120 min. Mary et al. [14] found that HAp with the same process degrades MB for 240 min with UV irradiation. HAp doped with metal semiconductor has been reported by Dewi et al. [13] by doping zinc oxide to the HAp for removal of MB and rhodamine B. Rocha et al. [22] synthesized gallium-containing HAp as a photocatalyst for the degradation of MB under 120 min of UV irradiation.

HAp was also synthesized by using natural sources. Fatimah et al. [19] has studied on synthesizing HAp derived from natural sources by using cockle shells as the CaCO_3_ source. The HAp was doped with TiO_2_ for photocatalytic degradation methyl violet under UV light irradiation. The synthesis of titanium-doped HAp using waste marble powder was done by Khan et al. [21] for the degradation of Congo red dye in wastewater. Another synthesis of HAp derived from natural sources by using mutton bone was done by Singh et al. [16]. HAp was doped with TiO_2_ photocatalyst for the degradation of MB. Yeasmin et al. [18] reported on the synthesis of HAp-TiO_2_-ZnO derived from eggshells as the photocatalyst for the degradation of MB and MO. Li and Meng [23] reported on the recent development of enhanced palladium (Pd) photocatalytic activity. Pd-TiO_2_ has been reported for the degradation of MB while Pd-ZnO was used as photocatalyst for the degradation of rhodium blue.

As a result, the objective of this research is to synthesize the HAp from clamshells by impregnation with Pd catalyst. The photoactive of Pd/HAp would next be investigated by its photocatalytic reduction of MO as a model of persistent water pollutants. The study’s novelty is based on a green synthesis strategy for producing HAp from clamshell biomaterials. Besides, loading of Pd does improve the photocatalytic efficiency where it shifts the adsorption profile of resulting Pd/HAp and creates photoactive-induced electron-hole pairs during the catalytic process. In this case, Pd acts as a sink on the surface of HAp for trapped electrons, hence slowing down the recombination of electron-hole pairs to ensure the stability of the photocatalyst during the photoreduction of methyl orange especially in extreme water conditions. The outcomes of this study suggest that HAp prepared using clamshell doped by Pd metal would be a safer, nontoxic, and potential candidate for the photodegradation of synthetic dyes used in textile and paper industries.

## 2. Materials and methods

Orthophosphoric acid (H_3_PO_4_, ≥ 85%), ammonia solution (NH_4_OH, ≥ 25%), and potassium iodide (KI, ≥ 99%) were acquired from Friedmann Schmidt. Potassium chloride (KCl, 99.5%) and ethanol (C_2_H_5_OH, 95%) were purchased from R&M. Palladium (II) chloride (PdCl_2_, 99%) and methyl orange (C_14_H_14_N_3_NaO_3_S, C.I. 13025) were obtained from Sigma Aldrich. In all experiments, deionized water (DI water, 18.2 MΩ·cm) was utilized.

### 2.1. Synthesis of HAp

The *Paphia undulata* clamshell was boiled before the contents were removed. To separate the contaminants, the clamshells were properly washed and cleaned with deionized water, then dried in an oven at 80 °C for 24 h. The raw materials were crushed after mechanical cleaning to create a fine calcium carbonate powder (CaCO_3_). Clamshells are made up of calcium carbonate (CaCO_3_) that has been calcined at 900 °C for 4 h, releasing carbon dioxide (CO_2_) to generate white calcium oxide (CaO).

HAp materials were synthesized by wet precipitation method. Calcium oxide (CaO) obtained from the clamshell was dissolved in deionized water to obtain 0.1 M of calcium hydroxide solution. It was stirred for about 6 h in order to achieve a uniform homogenous mixture of calcium hydroxide (Ca(OH)_2_). Subsequently, 0.6 M of H_3_PO_4_ was added dropwise into the Ca(OH)_2_ solution while stirring. The pH value of the solution was adjusted and varied (pH 8, pH 10, and pH 11.5) by adding 25% ammonia solution dropwise into the aforementioned solution. HAp nanoparticles were then sonicated into an ultrasonic bath for 60 min. After that, the solution was centrifuged at 5000 rpm for 5 min, and then washed three times with deionized water followed by ethanol. The washed precipitate was collected and dried using the freeze-drying method. Next, it was calcined at 700 °C for 2 h.

### 2.2. Synthesis of Pd precursor

Ten millilitres of an aqueous solution of potassium chloride (KCl) was added to 20 mL of an aqueous suspension of palladium (II) chloride (PdCl_2_) at water bath temperature. The resultant solution was then filtered off, and the filtrate was taken onto a porcelain basin. It was then evaporated to dryness. Next, the produced potassium tetrachloropalladate (II) (K_2_PdCl_4_) was dried in a calcium chloride desiccator at room temperature.

### 2.3. Impregnation of Pd/HAp catalysts

Pd/HAp catalyst was prepared by dispersing 1 g of HAp in 100 mL of deionized water containing 250 mg of coffee powder. Next, 100 mg of K_2_PdCl_4_ crystals were dissolved in deionized water and added dropwise to the solution as mentioned earlier under vigorous stirring. The mixture was stirred for 24 h at room temperature to produce HAp-supported Pd catalysts. The product obtained was washed with sufficient deionized water and filtered several times. Next, it was dried in an oven at 50 °C overnight.

### 2.4. Characterization of Pd/HAp catalysts

The prepared Pd/HAp samples were grounded into fine powders and placed into a sample holder. The sample holder was then placed in the sample stage of PANalytical Empyrean X-ray Diffractometer (XRD) (Almelo, Netherlands), which was equipped with anode Cu Kα (λ = 1.5406 Å) radiation to start the analysis. The analysis was carried out at a scan rate of 0.02° min^−1^ over a 2θ range of 0°–80° under 40 kV/40 mA. The crystallite size was calculated from XRD data Scherrer approximation according to:


(Equation 1) $$\text{D}=\frac{\text{K}\lambda }{\beta \text{cos}\theta },$$

where D is the crystallite size, K is the broadening constant, λ is the radiation wavelength (nm) from measuring the full width at half maximum of peaks (β), and θ corresponds to the peak position. Particle sizes in suspension of the catalysts were determined using a zeta-sizer nano series by Malvern technique with laser light diffraction. Next, 0.01 mg of catalyst was added into 10 mL deionized water and sonicated for 10 min. The sample was transferred into a disposable cuvette for size measurement at room temperature. For the X-ray fluorescence (XRF) analysis, the samples were grounded into fine powders and placed into the sample holder of Shimadzu Fluorescence Spectrophotometer microEDX-1400 (Tokyo, Japan). The analysis was carried out with a measurement diameter of 50 μm. The presence of Pd in Pd/HAp was analysed through inductively coupled plasma mass spectrometry (ICP-MS) analysis (Agilent 7500, USA). The Pd/HAp catalyst was dissolved in nitric acid, diluted with DI water 1000 times and filtered through 0.20 μm filter. PdCl_2_ was used as the standard solution for the analysis. The features of functional groups of Pd/HAp samples were analysed on a PerkinElmer Spectrum 400 Fourier Transform Infra-Red (FTIR) Spectrophotometer (Massachusetts, USA) in a scanning range of 500–4000 cm^−1^ using attenuated total reflection (ATR) method. For the UV-Vis analysis, the Pd/HAp samples were placed in a cuvette 34 holder of Perkin Elmer Lambda 35 UV/Vis Spectrophotometer (Massachusetts, USA) to begin analysis with the absorption range of 190–1100 nm. In UV-Vis spectroscopy, the main components are a light source, a monochromator for separation of polychromatic light into a range of individual wavelengths where each of their narrow bands was selected, a sample holder, and a detector. The wavelength from the UV-Vis was calculated to obtain the energy and the absorbance data can calculate the absorbance coefficient, α. A graph of α(hν)n against energy (eV) or also known as Tauc’s plot was plotted to obtain the optical bandgap of Pd/HAp from the UV-Vis spectrum based on Equation 2 below:


(Equation 2) $$\alpha (h\nu )n=K\;(h\nu -E_{g}),$$

where α is the absorption coefficient, hν is the incident photon energy, K is the energy-independent constant, E_g_ is the optical bandgap energy of nanoparticles, and n is the nature of transition. The optical bandgap energy of Pd/HAp can be obtained from the tangent line of the curve at α = 0 at the point where it intercepts the x-axis.

### 2.5. Experiments for MO photoreduction

The photoreduction of synthesized Pd/HAp samples (pH 8, pH 10, and pH 11.5) was evaluated through the degradation of MO as a model azo dye. 100 mg L^−1^ of the catalyst was dispersed into 10 mg L^−1^ of MO solution in a quartz tube with 40 mm outer diameter, and with inner diameter at (36 × 165) mm in length. The solution was then stirred in the dark until it reached the adsorption-desorption equilibrium between MO dye and the catalyst for 15–30 min. The photocatalytic reaction of the samples was initiated under UV irradiation and turned on at the same time from 95 W Hg-lamp (Atlantic Ultraviolet Corporation, UV-C Germicidal) equipped with cut-off wavelength ≥ 254 nm. After each 15-min contact time, a small amount of the mixture was taken out and placed into a cuvette. The solution was left to rest overnight for the catalyst to settle down. The photodegradation of MO solution was then analysed using a Perkin Elmer Lambda 35 UV/Vis Spectrophotometer (Massachusetts, USA) at λ_max_ = 464 nm. The influence of pH values of HAp in Pd/HAp on MO degradation was carefully examined. The degradation efficiency of MO using Pd/HAp was calculated by the following Equation 3:


(Equation 3) $$D_{e}=\frac{C_{o}-C_{f}}{C_{o}}\times 100\%,$$

where D_e_ is the removal efficiency, and C_o_ (mg L^−1^) and C_f_ (mg L^−1^) are the initial and final concentrations of MO solution before and after degradation, respectively.

## 3. Results and discussion

### 3.1. Characterization of Pd/HAp catalyst

**Figure 1:**
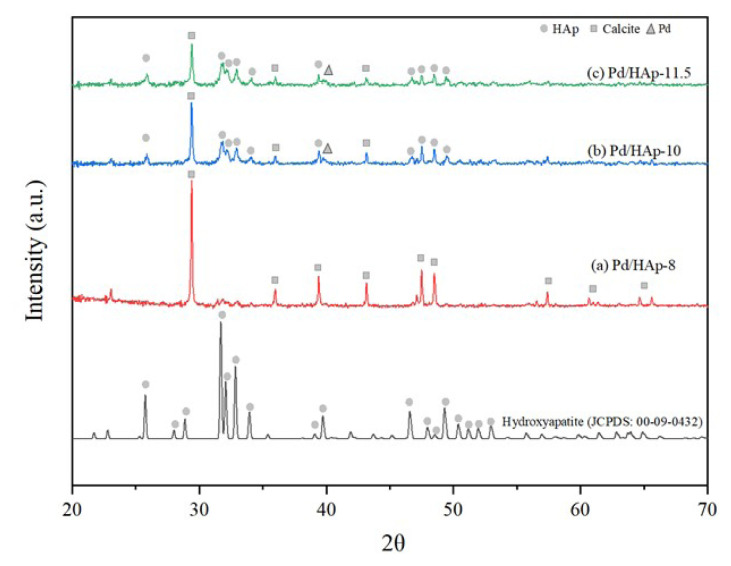
XRD diffractogram for Pd/HAp samples with different pH values of HAp at (a) pH 8, (b) pH 10, (c) pH 11.5

**Figure 2:**
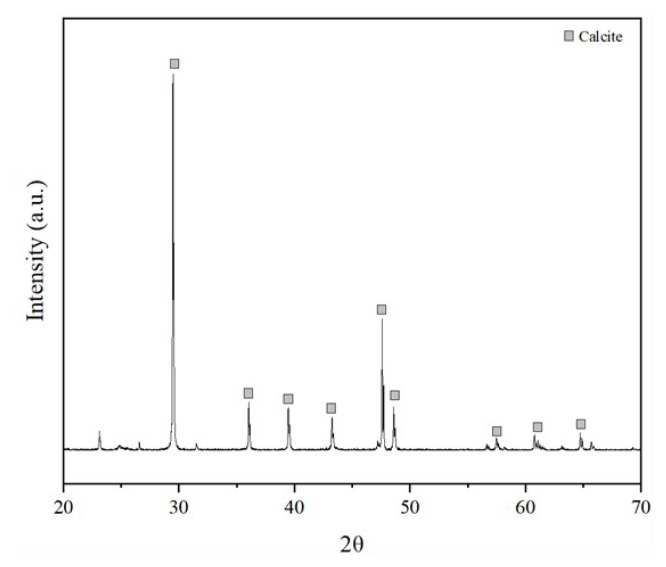
XRD pattern for calcium carbonate at calcite phase (JCPDS:00-05-0586).

XRD analysis was carried out to examine the phases and crystallographic structure of Pd/HAp samples. Figure 1 shows that the XRD analysis of Pd/HAp catalysts of different pH values. From Figure 1a, Pd/HAp-8 has almost the same XRD characteristic peak as calcite as shown in Figure 2 (JCPDS:00-05-0586). The intense peaks confirmed the polycrystalline nature of HAp. The peak at 25.1° for HAp is absent for Pd/HAp-8. Compared to Pd/HAp for pH 10 and 11.5, Pd/HAp-8 has the highest calcite peak at 29.4°. Pd/HAp-8 also exhibited the lowest peak intensity around 2θ of 32° to 35° compared to pH values of 10 and 11.5. This is due to the influence of pH towards the formation of HAp. The decrease in pH decreases the saturation state by shifting the equilibrium of phosphate species from H_3_PO_4_ to ${\text{PO}}_{4}^{3-}$, indicating that the ${\text{PO}}_{4}^{3-}$ was insufficient to form HAp in pH 8. This finding is similar to those of the previous reports by Gally et al. [24].

The XRD patterns of Pd/HAp pH 10 and 11.5 as shown in Figures 1b and 1c have common peaks consisting of the main characteristic peaks of HAp according to JCPDS 00-09-0432. It shows that Pd doping insignificantly affects the crystalline structure of HAp at pH 10 and 11.5. Pd/HAp-11.5 has HAp peak at 10.3° as HAp compared to Pd/HAp-10. However, there are additional peaks observed at 2θ of 29.4°, 35.1°, 38.2°, and 42.7° for both XRD patterns indicating the presence of calcite according to the JCPDS pattern of calcite in Figure 2. The characteristic peak of Pd appears at 2θ = 40.11° for Pd/HAp-10 and Pd/HAP-11.5 [25]. Moreover, the relative intensities of these peaks for Pd-doped HAp samples were reduced in comparison to Hap, which indicates that the crystallinity of HAp is reduced as Pd-doped into the support. This has been described in previous studies at which HAp owns a hexagonal crystal structure, but the introduction of a metal into the system results in system asymmetry. Therefore, the crystallinity of the material is decreased, leading to reduced intensity peaks [26, 27].

The crystallite size of Pd/HAp calculated from the XRD data using Scherrer equation from Equation 1 above. The results revealed that the crystallite sizes of Pd/HAp are at 124.65 nm, 44.07 nm, and 31.51 nm for HAp synthesized at pH 8, 10, and 11.5, respectively, as shown in Table 1.

**Figure 3:**
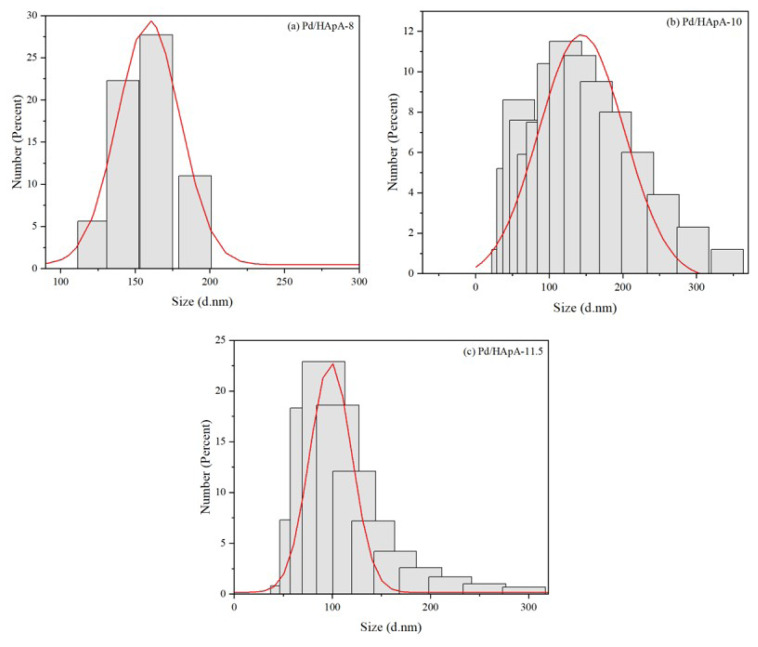
Particle size distribution for (a) Pd/HAp-8, (b) Pd/HAp-10, and (c) Pd/HAp-11.5.

The particle size distribution of Pd/HAp was analysed using zeta-sizer nano-series as shown in Figure 3. Figure 3a shows the particle size distribution for Pd/HAp-8 with the range of 120–200 nm with polydispersive index (PDI) of 1.000. The mean size of Pd/HAp-8 is at 157.4 nm. The particle size distribution for Pd/HAp-10 is shown in Figure 3b with sizes of 40–470 nm. The mean size is 153.7 nm with PDI at 0.982. Figure 3c shows that particles for Pd/HAp-11.5 are more homogenous in terms of size with one sharp peak of nano range between 25 and 300 nm with mean size at 121.8 nm. Researchers have reported that low particle size of catalyst enhanced its photocatalytic activities due to large surface area of light adsorption [28].

XRF analysis, on the other hand, was performed to study the chemical compositions of minerals that are present in the Pd/HAp samples as well as its calcium-to-phosphorus (Ca/P). Table 2 shows the Ca/P ratio of Pd/HAp samples with different pH values. It is observed that the Ca/P ratios of all samples are within the range of 1.6 to 1.8 except for pH 11.5. The expected value of Ca/P ratio is slightly lower than 1.67 due to the ionic substitution of Pd(II) ions into Ca(II) ions in the crystal lattice, which reduced the composition of Ca as compared to P.

According to XRF analysis for Pd/HAp, along with the main element characteristics for HAp, which are Ca, P, and O, Pd was detected only for Pd/HAp at pH values of 10 and 11.5. To confirm this, ICP-MS was done to analyse the presence of Pd. The results show the presence of Pd at 11.40% and 12.01% for Pd/HAp synthesized at pH 10 and 11.5, respectively. The absence of Pd at HAp synthesized at pH 8 might be due to the leaching of Pd metal through washing which remarks a low affinity of Pd on the surface of HAp.

**Figure 4:**
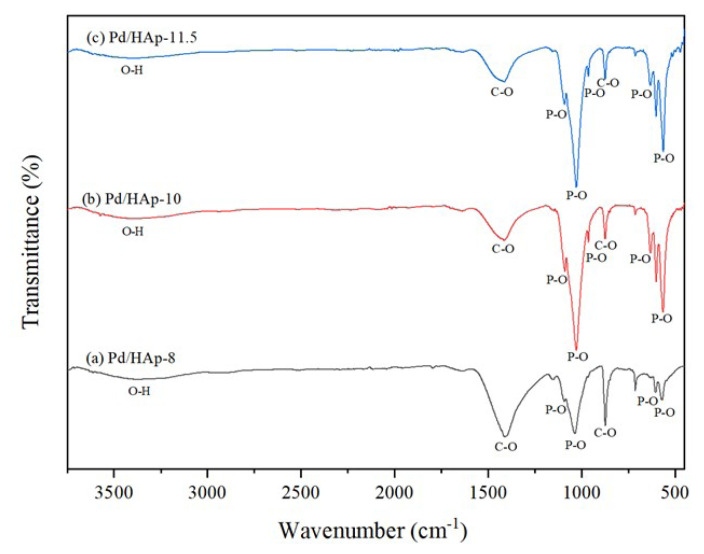
FTIR sSpectrum of Pd/HAp at different HAp pH values; (a) pH 8, (b) pH 10, and (c) pH 11.5.

The functional groups of Pd/HAp samples of different pH values were investigated using FTIR spectra as illustrated in Figure 4. The FTIR spectra obtained for Pd/HAp of different pH values show similar shape and most characteristic peaks. Table 3 shows the FTIR assignment of Pd/HAp. The most intensive band around 1030 cm^−1^ is assigned to the antisymmetric of P-O stretching vibrational mode of ${\text{PO}}_{4}^{3-}$ group. Furthermore, the absorption peak around 964 cm^−1^ indicates the symmetric vibration of P-O bond. The bands in the range of 570 to 636 cm^−1^ are assigned to the vibrational bending mode (v4) of O-P-O bonds of ${\text{PO}}_{4}^{3-}$ group. Based on the previous studies done on HAp photocatalytic mechanisms, ${\text{PO}}_{4}^{3-}$ group would have played a major and important role in the dye degradation reaction [15, 29]. Besides, the broad peak obtained at absorption wavenumber around 3300 cm^−1^ indicates the presence of OH^−^ groups. As for the vibration of carbonate groups, the peaks were observed around 874 cm^−1^ and 1413 cm^−1^ and it was absorbed from the ambient atmosphere [27, 30].

At these absorption peaks, it shows that the carbonate groups substituted at phosphate position which is known as B-type. This substituted carbonate affects the thermal stability as well as the degradation rate [31]. Other than that, there was a difference in intensity between some peaks in Pd/HAp samples. It was observed that the intensities of absorption peaks for both Pd/HAp samples at pH value of 10 and 11.5 are similar. However, it is not the case for Pd/HAp sample at the pH value of 8 where the intensity of the absorption peak is lower at 1030 cm^−1^, 964 cm^−1^, and in the range of 570 to 636 cm^−1^. This may agree with the result obtained in XRD analysis where the pH influences the formation of HAp due to the ${\text{PO}}_{4}^{3-}$ groups production.

**Figure 5:**
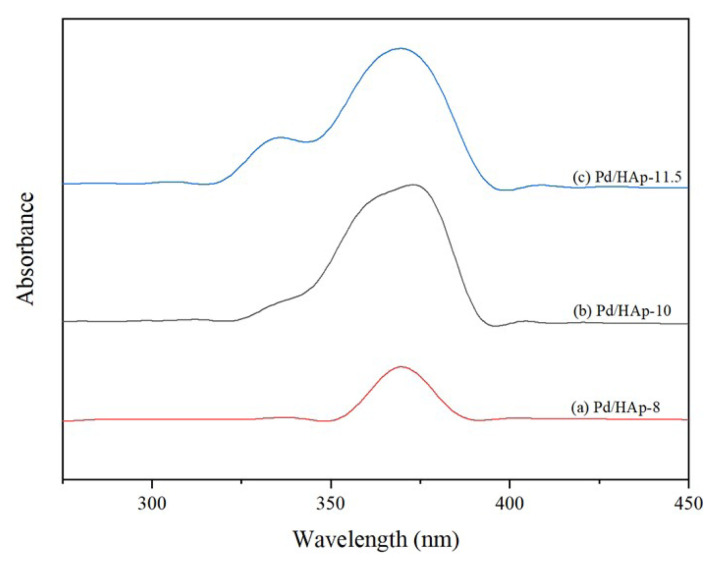
UV-Vis absorption spectra of (a) Pd/HAp-8, (b) Pd/HAp-10, and (c) Pd/HAp-11.5.

Figure 5 shows the absorption curve of Pd/HAp at different pH. There are absorption bands observed at 370 nm for all the photocatalysts. As the pH of Pd/HAp increases, the absorbance peak increases. Other than that, another peak of 334 nm appears at the UV spectra of Pd/HAp-10 and 11.5 corresponding to the presence of [PdCl_4_]^2−^ ion [32]. Hence, both Pd/HAp-10 and 11.5 contain Pd ion from the UV-Vis result.

**Figure 6:**
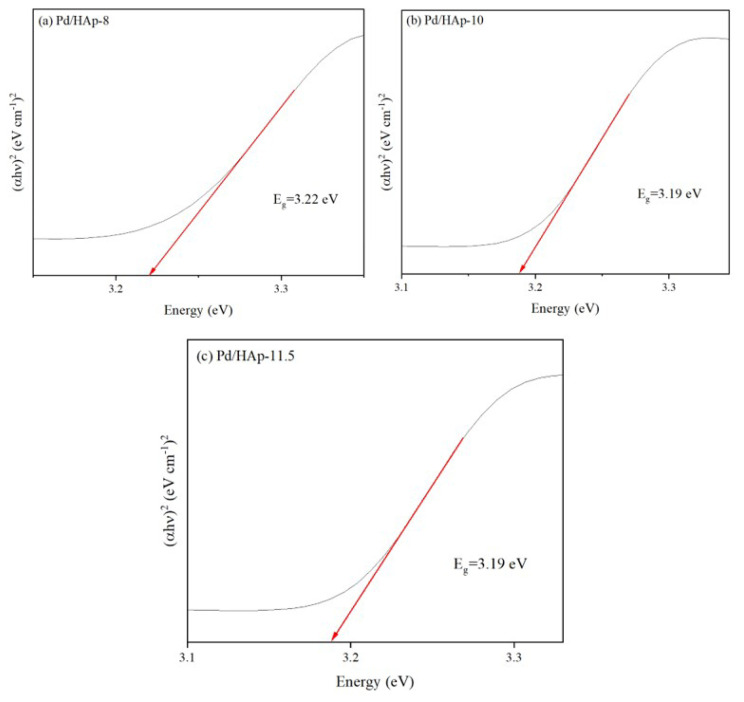
Bandgap calculated using Tauc’s plot from UV-Vis spectroscopy; (a) Pd/HAp-8, (b) Pd/HAp-10, and (c) Pd/HAp-11.5.

The bandgap (E_g_) curve of Pd/HAp with different pH is estimated from Tauc’s plot as shown in Figure 6. The bandgap of the Pd/HAp catalysts are all above 3 where it is photoactive at UV region. The band gap of Pd/HAp-8, Pd/HAp-10, and Pd/HAp-11.5 are 3.22 eV, 3.19 eV, and 3.19 eV, respectively. The bandgap values obtained decreases with respect to increasing pH value, which is due to the formation of more PO_3_^4−^ ion on lattice of HAp.

### 3.2. Photocatalytic activities of Pd/HAp towards reduction of MO

**Figure 7:**
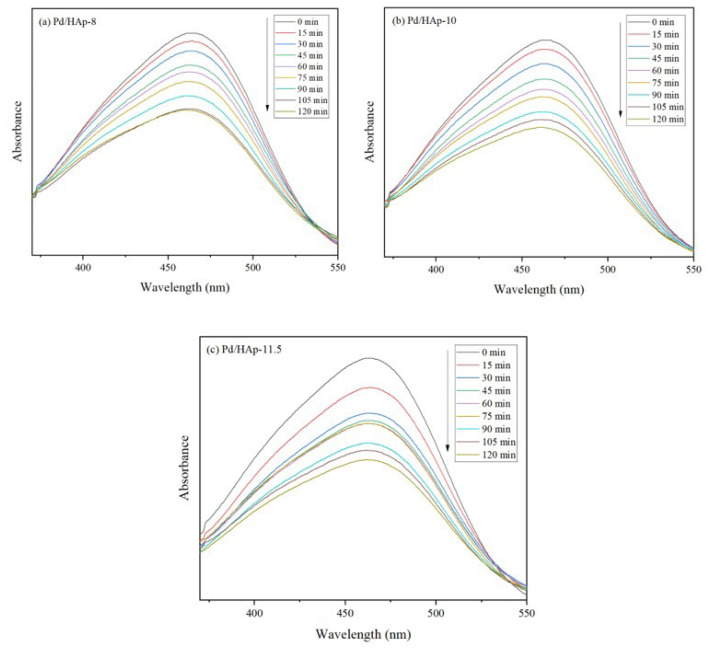
UV-Vis absorption spectra of Pd/HAp samples for 120 min at (a) pH 8 (b) pH 10 (c) pH 11.5.

In order to determine the photocatalytic activities of Pd/HAp catalyst with different pH values of HAp, the photodegradation of a dye which was MO was used as model azo dye was evaluated. Throughout the experiment, the conditions were fixed at 0.1 g (catalyst loading), 10 mgL^−1^ (initial dye concentration), and 95 W UV lamp (light intensity). The photodegradation of MO was determined by the intensity of absorption band of MO. The solution was first stirred in the dark until it reached the adsorption–desorption equilibrium between MO dye and the catalyst. The adsorption of Pd/HAp synthesized at pH 8, 10, and 11.5 reached 14.3%, 17.2%, and 11.3%, respectively. Figure 7 demonstrates the UV spectra of photodegradation of MO using Pd/HAp samples. MO shows the maximum absorption peak at 464 nm and that indicates the colour risen from chromophore with an azo group [2–4]. The appropriate adsorption-desorption was conducted under dark condition and it was observed that there were declining trends between adsorptions.

The calculated degradation, (D_e_) of Pd/HAp samples reached its highest value of 41.63%, 48.17%, and 43.64% after 120 min of continuous UV irradiation for pH values of 8, 10, and 11.5, respectively. This result agreed on the XRF analysis above where the presence of Pd could increase the photocatalytic performance as Pd/HAp of pH 10 had the highest degradation efficiency. However, even without Pd content in HAp, it could also photodegrade MO under UV irradiation. This is probably due to the presence of ${\text{PO}}_{4}^{3-}$ ions in HAp lattice. It has been proposed that the photocatalytic behaviour of HAp is due to a shift in the electronic state of the surface phosphate group under UV irradiation due to the production of active superoxide anion radicals [24, 32, 33]. Therefore, it explained the photodegradation of MO using Pd/HAp of pH values of 8 and 11.5. On the other hand, based on the Langmuir–Hinshelwood model, the degeneration of dye molecules is found to obey pseudo-first-order kinetics [14, 15]. These results correspond with the result obtained by Al-Ahmed et al., which also obeys pseudo-first-order degradation on the removal of MB [34].

**Figure 8:**
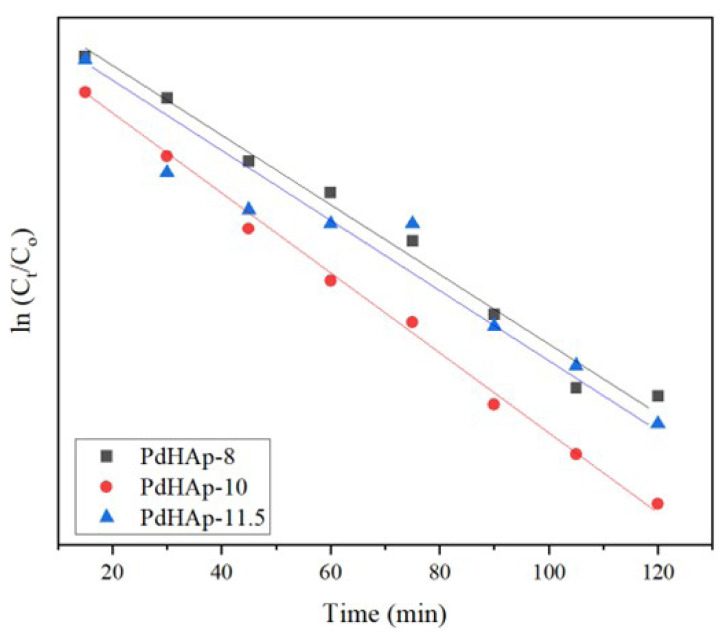
A plot of ln C_t_/C_o_ versus time for different Pd/HAp samples obtained at various pH values.

Figure 8 shows the plot of ln C_t_/C_o_ versus time for different Pd/HAp samples obtained at various pH values, where K_app_ = slope corresponding results are depicted in Table 4. As all samples exhibited high correlation coefficients of R^2^ ≥ 0.95 except for Pd/HAp at pH value of 11.5, the photodegradation reactions were said to obey pseudo-first-order kinetics.

As the light-irradiated energy was greater than the band gaps of Pd/HAp samples, it was possible to produce electron-hole pairs. The photoelectrons excite to the conduction band (CB), whereas the conjugated hole moves in the valence band (VB). Radicals such as hydroxyl radicals that are formed from the oxidation of water molecules and superoxide anions (O^2−^) that are formed from the reduction of oxygen molecules are responsible for degrading the targeted dye. Direct oxidation of the organic dye pollutants may also occur on the surface of the catalyst [2–4]. Based on the natural source HAp-based catalyst study, the photocatalysis using Pd/HAp synthesized at pH 10 demonstrated photodegradation efficiency at 48%, which is higher compared to the photocatalysis performed by Yeasmin et al. [18] using HAp-TiO_2_-ZnO that demonstrated photodegradation efficiency at 45% using a similar methyl orange dye under UV irradiation. Palladium has a high absorption coefficient in the UV range, which makes it an excellent candidate for photocatalysis. This property allows palladium to absorb a large amount of light energy and efficiently convert it into chemical energy, which can be used to drive chemical reactions [23].

#### 3.2.1. Recyclability test

**Figure 9:**
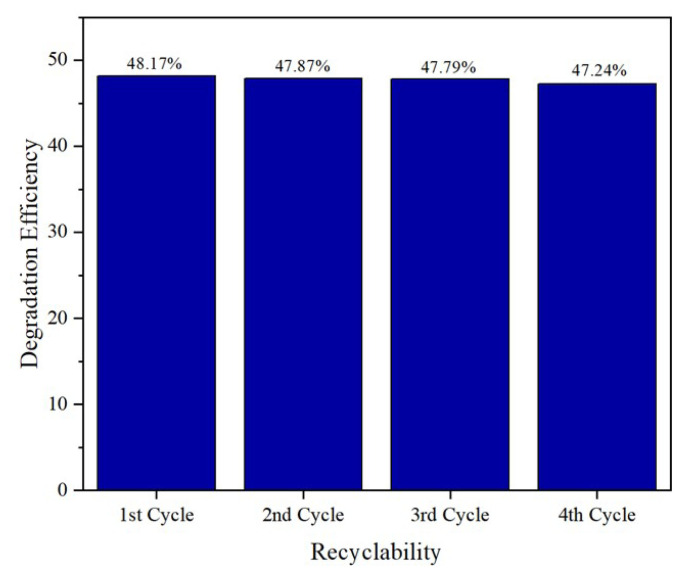
The comparison of catalyst recyclability of Pd/HAp synthesized at pH 10.

The stability and reusability of Pd/HAp synthesized at pH 10 were done four times. The spent catalyst was washed and dried in an oven at 70 °C. The following reaction was performed using freshly prepared MO with the same condition as the first photoactivity measurement. Figure 9 represents the comparison of the catalyst’s first, second, third, and fourth catalytic potentials towards MO degradation under UV irradiation. The degradation efficiency of Pd/HAp synthesized at pH 10 with the range at 48.17%–47.24% after fourth use. Since the difference in the degradation efficiency after every run is less than 1%, it is suggested that no significant reduction in photodegradation efficiency was observed. Palladium is stable under photocatalytic conditions, which means that it can remain active for extended periods without undergoing degradation or deactivation. This stability is crucial for the long-term performance of photocatalytic reactions [23]. It can be concluded that Pd/HAp synthesized at pH 10 exhibits optimum stability and the possibility to be recycled and reused.

## 4. Conclusion

In this study, the Pd substituted with different pH values of HAp via wet precipitation method was successfully synthesized and applied as a heterogeneous catalyst for the photodegradation of MO under UV irradiation for a contact time of 120 min. The characterization results showed that HAp samples produced with the Ca/P molar ratios deviated from the literature value of 1.67, indicating Ca is sufficient HAp material. The photoreduction of MO by Pd/HAp demonstrated that the degradation efficiency reached its highest value of 41.63%, 48.17%, and 43.64% after 120 min of continuous UV irradiation for Pd/HAp with pH values of 8, 10, and 11.5, respectively. With the bandgap of 3.22 eV for Pd/HAp-8 and 3.19 eV for both Pd/HAp-10 and 11.5, the obtained outcomes of this study revealed the potential of biomaterials based on HAp to be photoactive with Pd metals to find a new application in the reduction of persistent water pollutants. This study opens a new insight and opportunity on application of HAp as low-cost waste materials for advanced application of water treatment.

## Figures and Tables

**Table 1:** Crystallite size calculated from XRD measurement.

Pd/HAp synthesized at pH	Mean Crystallite size (nm)
8	124.65
10	44.07
11.5	31.51

**Table 2:** Ca/P ratio of Pd/HAp photocatalyst with different pH values of HAp.

pH of HAp	Ca/P ratio	Pd wt (%)
8	1.63	-
10	1.77	11.40
11.5	1.44	12.01

**Table 3:** FTIR assignment of Pd/HAp.

Pd/HAp-8	Pd/HAp-10	Pd/HAp-11.5	Assignment
-	480.16	-	v2 of O-P-O
568.12	563.36	562.6	v4 of ${\text{PO}}_{4}^{3-}$
604.36	599.65	600.05	
628.13	630.05	630.50	
871.93	872.79	827.8	v2 of ${\text{CO}}_{3}^{2-}$
-	963.08	963.09	P-O bond
1034.83	1027.07	1026.55	v3 of ${\text{PO}}_{4}^{3-}$
1090.26	1088.68	1089.99	P-O stretching of ${\text{PO}}_{4}^{3-}$
1148.78	1146.55	1155.03	Degenerate stretching of ${\text{HPO}}_{4}^{2-}$
1408.90	1413.59	1413.18	v3 of ${\text{CO}}_{3}^{2-}$
1636.53	1636.27	1636.28	Bending mode of O-H
2114.88	2097.29	-	Overtone and combination of ${\text{PO}}_{4}^{3-}$
-	2320.65	-	
3368.03	3392.07	3381.1	O-H stretching

**Table 4:** The photodegradation efficiency (%) and pseudo-first-order constant include Kapp (min^−1^) and R^2^ for Pd/HAp samples according to their ability to remove MO dye from aqueous solutions.

pH of Pd/HAp	Photodegradation efficiency (%)	K (min^−1^)	R^2^
8	41.6	0.0036	0.9862
10	48.2	0.0041	0.9962
11.5	43.6	0.0032	0.9388
